# TGF-β1 Inhibition, Cytokine Modulation, and Clinical Response to Seleno-L-Methionine Plus Axitinib and Pembrolizumab in Treatment-Naïve Clear Cell Renal Cell Carcinoma

**DOI:** 10.3390/ijms27219825

**Published:** 2026-11-02

**Authors:** Matthew R. Kroll, Yousef Zakharia, Fernando Maciel Barbosa, Vitor Fiorin de Vasconcellos, John M. Rieth, Mohammed Milhem, Mariana Pilon Capella, Youcef M. Rustum

**Affiliations:** 1 Division of Hematology, Oncology and Blood and Marrow Transplantation, Department of Internal Medicine, University of Iowa, Iowa City, IA 52242, USA; fernando-macielbarbosa@uiowa.edu (F.M.B.); vitor-vasconcellos@uiowa.edu (V.F.d.V.); john-rieth@uiowa.edu (J.M.R.); mohammed-milhem@uiowa.edu (M.M.); mariana-piloncapella@uiowa.edu (M.P.C.); youcef.rustum@roswellpark.org (Y.M.R.); 2 Division of Hematology and Medical Oncology, Mayo Clinic Arizona, Phoenix, AZ 85054, USA; zakharia.yousef@mayo.edu; 3 Roswell Park Comprehensive Cancer Center, Buffalo, NY 14263, USA

**Keywords:** clear cell renal cell carcinoma, seleno-L-methionine, TGF-β1, hypoxia-inducible factor, axitinib, pembrolizumab, immune checkpoint inhibitor, VEGF, cytokines, vascular normalization

## Abstract

Metastatic clear cell renal cell carcinoma (ccRCC) remains largely incurable, and the efficacy of pembrolizumab plus axitinib may be limited by hypoxia-associated signaling, immunosuppressive cytokines, and metabolic reprogramming involving the TGF-β1/HIF axis. Seleno-L-methionine (SLM) is a pharmacologic inhibitor of this axis and its downstream effectors, including PD-L1 and VEGF. In a phase I/II pilot study (ClinicalTrials.gov NCT05363631), patients with newly diagnosed metastatic ccRCC received SLM twice daily for 14 days, then once daily with standard-dose axitinib; pembrolizumab was added on day 15. Endpoints included circulating (plasma) TGF-β1, plasma selenium, circulating cytokines, efficacy, and safety. Twelve patients were treated and evaluable for safety (six at 5000 µg, six at 6000 µg SLM); ten were evaluable for response. The objective response rate was 70% (complete response 40%, partial response 30%). Of the four patients with CR, two had favorable and two had intermediate International Metastatic Renal Cell Carcinoma Database Consortium disease. Grade 3 hypertension was documented in 58.3%, about twice the pivotal-trial rate, without treatment discontinuations or new toxicities. SLM increased plasma selenium and produced time-dependent inhibition of TGF-β1 and modulation of immune-related cytokines (CXCL13, IFN-γ, TNF-α, IL-6, IL-8). In this pilot study, SLM combined with axitinib and pembrolizumab showed time-dependent TGF-β1 inhibition, cytokine modulation, and encouraging antitumor activity without treatment-limiting toxicity, supporting further investigation of SLM for targeting the TGF-β1/HIF-driven immunosuppressive microenvironment in ccRCC.

## 1. Introduction

Clear cell renal cell carcinoma (ccRCC) is characterized by constitutive activation of the HIF-1α/HIF-2α pathway, primarily resulting from VHL loss, together with marked overexpression of TGF-β1. These pathways contribute to an immunosuppressive, hypoxic, angiogenic, and metabolically reprogrammed tumor microenvironment that limits durable responses to immune checkpoint inhibitor (ICI)-based therapy [1,2,3]. Although pembrolizumab plus axitinib has become a recommended first-line regimen for metastatic ccRCC, most patients ultimately develop disease progression, underscoring the need for strategies that modify the tumor microenvironment and overcome intrinsic and acquired resistance [4,5].

Pharmacologic-dose Seleno-L-methionine (SLM) represents a potential tumor-conditioning strategy because it targets multiple mechanisms implicated in ccRCC therapeutic resistance [6,7,8,9,10,11]. Preclinical studies indicate that SLM suppresses TGF-β1 and promotes prolylhydroxylase 2-mediated, VHL-independent degradation of HIF-1α and HIF-2α, thereby potentially disrupting the signaling networks that sustain hypoxia, angiogenesis, immune evasion, and metabolic reprogramming [6,7,8,9,10,11]. These effects provide a mechanistic rationale for evaluating SLM as a priming agent before and during VEGFR inhibition and PD-1 blockade.

We hypothesize that pharmacologic-dose SLM administered before and concurrently with axitinib and pembrolizumab reprograms the TGF-β1/HIF-driven immunometabolic and cytokine networks of ccRCC, thereby converting an immunosuppressive tumor microenvironment into one more permissive to antitumor immune responses. The proposed studies will determine whether the SLM-mediated inhibition of TGF-β1/HIF signaling is associated with modulation of cytokines, metabolic pathways, and immune-response biomarkers and whether these changes correlate with clinical response to axitinib plus pembrolizumab.

The sequential treatment strategy is therefore designed as biologic tumor priming rather than simply combining three agents. SLM is intended to first disrupt key resistance pathways, axitinib to subsequently inhibit VEGFR-dependent angiogenesis within the remodeled tumor microenvironment, and pembrolizumab to enhance antitumor T-cell activity. The proposed mechanistic and translational studies will test whether this sequence improves the depth and durability of response in previously untreated patients with metastatic ccRCC.

## 2. Results and Discussion

### 2.1. TGF-β1 Is a Druggable Target of Pharmacologic-Dose SLM

**Figure 1:**
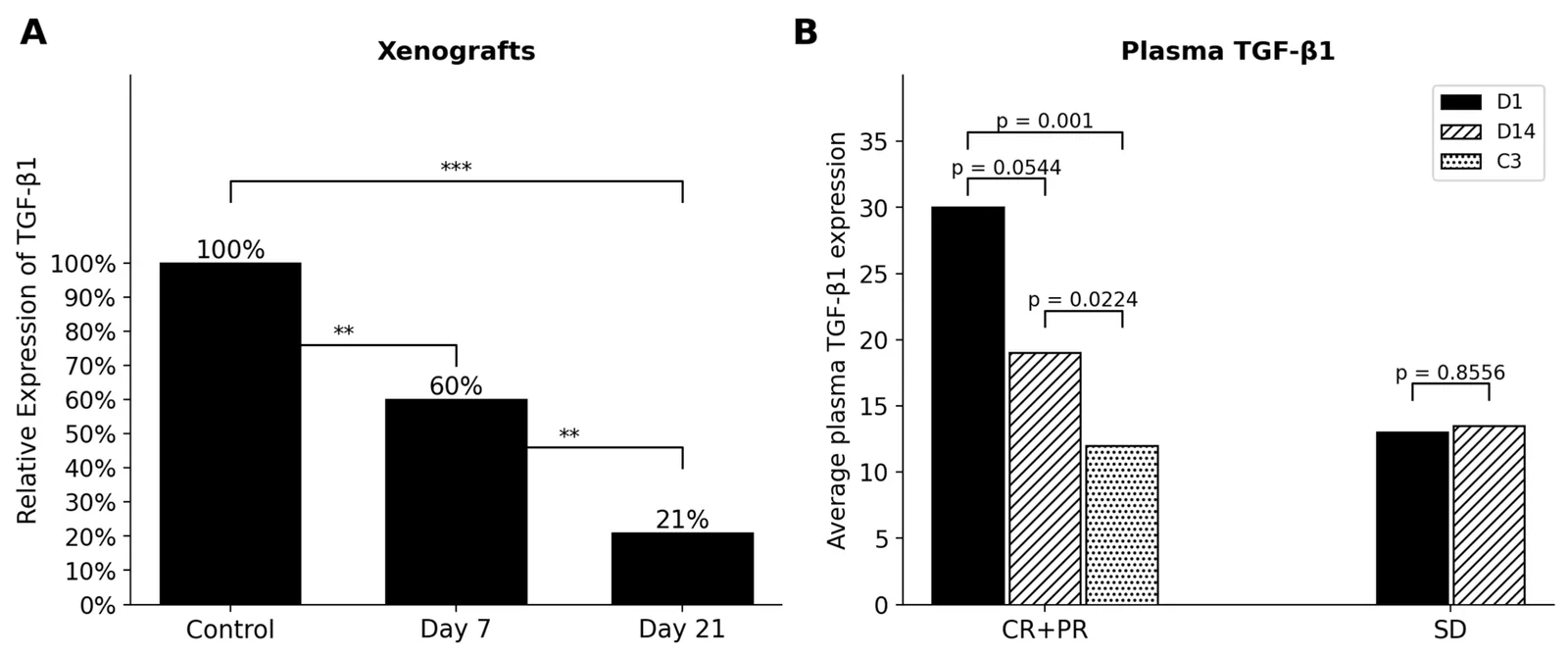
In vivo downregulation of TGF-β1 by pharmacologic dose of SLM is time dependent in 786-0 xenografts treated with 10 mg/kg/dx21 (**A**) and in ccRCC patients treated with 4000 mcg bidx14 days then daily with axitinib for more than 3 months (**B**). In panel (**A**), xenograft tumor-tissue TGF-β1 was analyzed by Western blot as previously described [6], with tumors obtained on days 7 and 21. In panel (**B**), circulating (plasma) TGF-β1 was measured in patients at day 14 (SLM alone) and after approximately 3 months of SLM plus axitinib and pembrolizumab. CR, complete response; PR, partial response; SD, stable disease. **, *p* < 0.01; ***, *p* < 0.001.

Inhibition of overexpressed TGF-β1 in 786-O ccRCC xenografts following treatment with a nontoxic pharmacologic dose of SLM (10 mg/kg/day, orally) was time-dependent, with significant inhibition observed by day 7 and greater suppression by day 14 **(**Figure 1A). We previously demonstrated in xenograft models that HIF-1α and HIF-2α are also druggable targets of therapeutic-dose SLM [6,10,11]. Concurrent inhibition of TGF-β1 and HIF signaling was associated with enhanced antitumor activity of multiple chemotherapeutic agents and biologically targeted therapies [12].

We further evaluated circulating TGF-β1 in patients with ccRCC receiving pharmacologic-dose SLM (4000 µg twice daily for 14 days, followed by once-daily administration) in combination with axitinib (5 mg twice daily, initiated on day 14) for more than 3 months (Figure 1B). Consistent with the preclinical findings, TGF-β1 suppression was also time-dependent in patients achieving complete or partial responses. In contrast, patients with stable disease exhibited lower baseline TGF-β1 levels that showed little further modulation during SLM treatment.

Collectively, these preclinical and clinical findings support TGF-β1 as a pharmacologically druggable target of nontoxic, therapeutic-dose SLM and indicate that protracted treatment (SLM followed by its combination with axitinib and pembrolizumab) is associated with progressively greater TGF-β1 inhibition; because the later assessments occurred during combination therapy, the contribution of SLM alone cannot be isolated.

### 2.2. Plasma Selenium Concentrations in ccRCC Patients Are Dose Dependent

Dose escalation of SLM was evaluated in patients to confirm that the plasma selenium concentrations are demonstrated to be molecularly effective and therapeutically efficacious in clinal xenograft models and can be safely used with doses administered clinically without side effects. As shown in Figure S1, increased plasma selenium was SLM-dose-dependent, with the suitable selenium concentrations being achieved with SLM doses of 4000 mcg and higher. In the six patients treated with a 6000 mcg SLM dose, the dose was then administered twice daily for 14 days.

### 2.3. Modulation of Plasma Cytokines by SLM Treatment in ccRCC

In ccRCC, circulating cytokines may serve as dynamic indicators of the systemic inflammatory and immunologic milieu and may also reflect changes in the tumor microenvironment. Although baseline cytokine profiles may have prognostic value, longitudinal changes during treatment may provide additional insight into treatment-induced immune modulation and mechanisms associated with therapeutic response or resistance.

**Figure 2:**
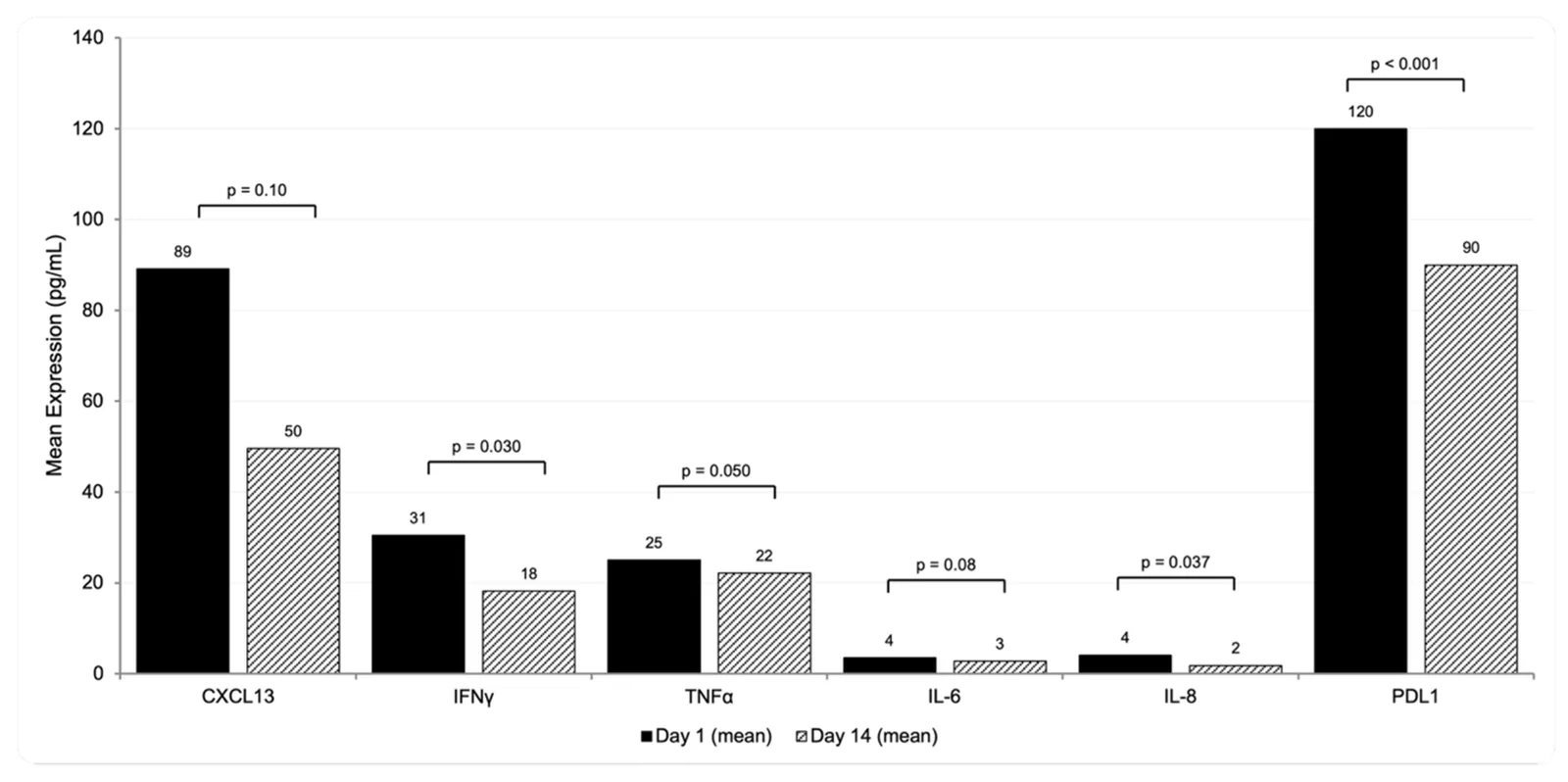
Effects of SLM on the expression levels of selected cytokines in ccRCC patients treated with the 6000 mcg SLM. analysis was carried out in plasma before therapy as baseline, day 1, and on day 14 at 2 h post SLM administration.

Figure 2 shows changes in plasma cytokine levels following treatment with SLM alone twice daily for 14 days. The pilot data demonstrate that pharmacologic-dose SLM modulates multiple circulating cytokines, including CXCL13, IFN-γ, and IL-8. These treatment-associated changes support a systemic immunomodulatory effect of SLM and are consistent with its proposed role in remodeling the inflammatory and immunosuppressive milieu in ccRCC. The direction and magnitude of change for each cytokine, together with baseline and day-14 values and statistical significance, are summarized in Figure 2.

Collectively, these findings provide preliminary evidence that short-term SLM treatment can alter the circulating cytokine profile in patients with ccRCC. Further studies in larger cohorts are required to determine whether the magnitude and pattern of cytokine modulation are associated with clinical response, resistance, or treatment-related toxicity.

### 2.4. Patients’ Demography and Adverse Events

A total of 12 patients started the study treatment between 19 September 2022 and 17 November 2025 and were included in the reported safety analysis. Six patients were treated at SLM doses of 5000 μg, and six patients were treated at 6000 μg. The 12 patients treated at these doses were included in the efficacy and safety analysis. The median time on treatment is 13.0 months (range 3.3–42.5 months) The representativeness of the trial population is summarized in Table 1. All participants in this trial were non-Hispanic Caucasian (12/12). In total, 10/12 (83.3%) patients were male. Median age was 71. By IMDC risk group, 4/12 (33%) of the patients were favorable, 7/12 (58.3%) were intermediate, and 1/12 (8.3%) was in the poor risk group. The most common grade 3 events were hypertension (58.3%), ALT increase (16.7%), abdominal pain (16.7%), acute kidney injury (16.7%), and diarrhea (16.7%). (Table 2). No grade 4 or 5 were seen. No more acute grade 3 nor delayed grade 3 hypertension was seen in the triple combination when compared to the previously reported SLM + axitinib doublet [7]. No treatments delays or stoppages were attributable to grade 3 hypertension.

During the initial 14-day priming phase of SLM monotherapy (before axitinib and pembrolizumab), treatment-emergent adverse events were infrequent, with 6 of 12 patients (50%) experiencing none. Events that did occur were predominantly grade 1 (dyspnea on exertion, fatigue, cough, dizziness, proteinuria, and hematuria); one patient had a grade 2 infusion-related reaction, and one grade 3 venous thromboembolism occurred on day 14 and was attributed to the underlying malignancy rather than to SLM. No selenosis-specific toxicities (e.g., garlic or metallic breath, nail or hair changes, selenium dermatitis, or peripheral neuropathy) were observed during SLM monotherapy, and whole-blood selenium reached the pre-specified target range (35–45 µM) by day 14 without selenium-attributable grade ≥ 3 toxicity. Adverse events increased in frequency only after the addition of axitinib and pembrolizumab on day 15 (Table 2).

### 2.5. Clinical Outcomes in Patients Treated with SLM Triple Combination

**Figure 3:**
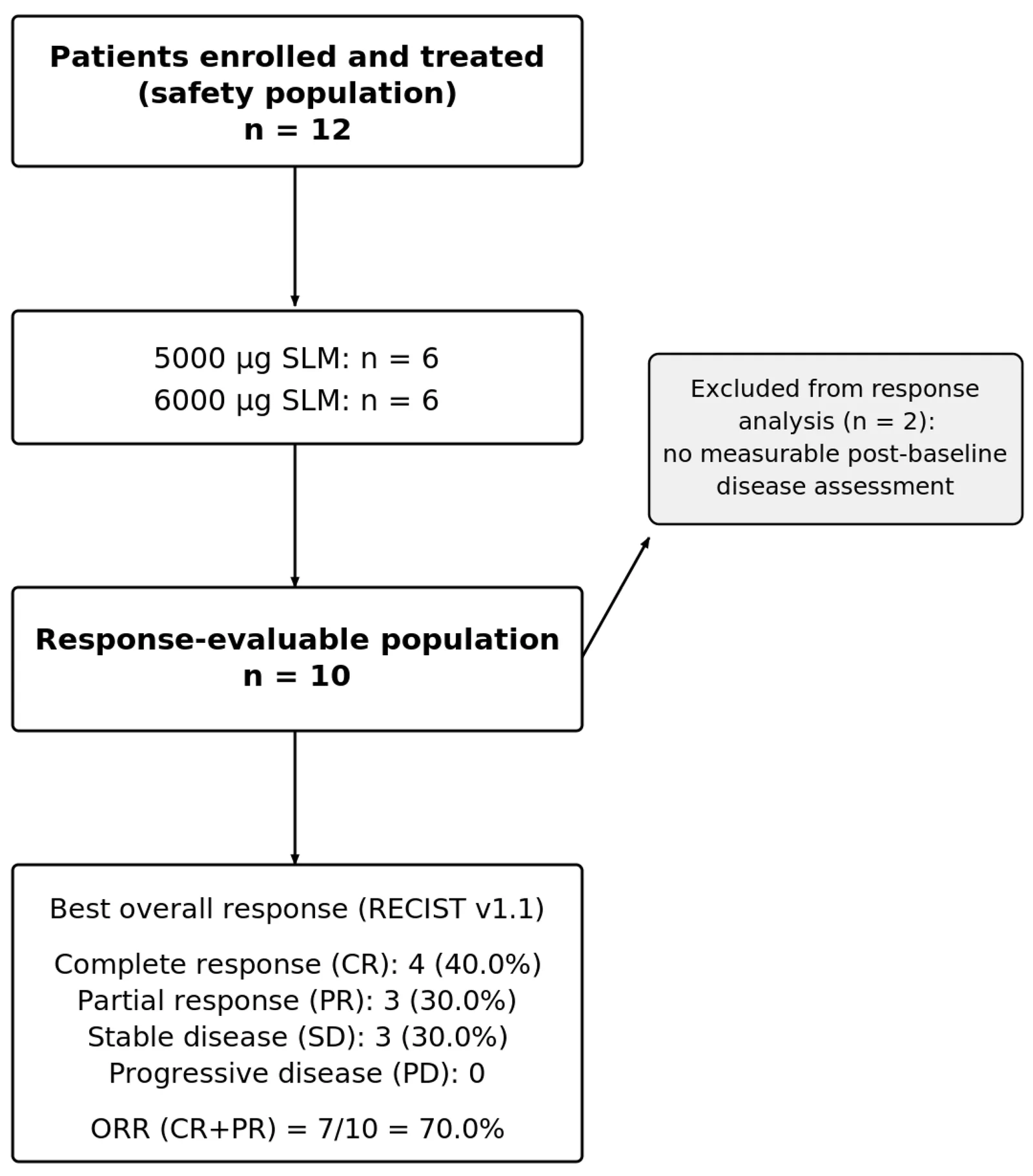
Consort diagram of patient disposition. Twelve patients were treated and included in the safety population (6 at 5000 µg and 6 at 6000 µg SLM). Two patients without measurable post-baseline disease assessment were excluded from the response analysis, leaving 10 response-evaluable patients. Best overall response by RECIST v1.1: complete response 4 (40.0%), partial response 3 (30.0%), stable disease 3 (30.0%), and progressive disease 0; objective response rate 7/10 = 70.0%.

**Figure 4:**
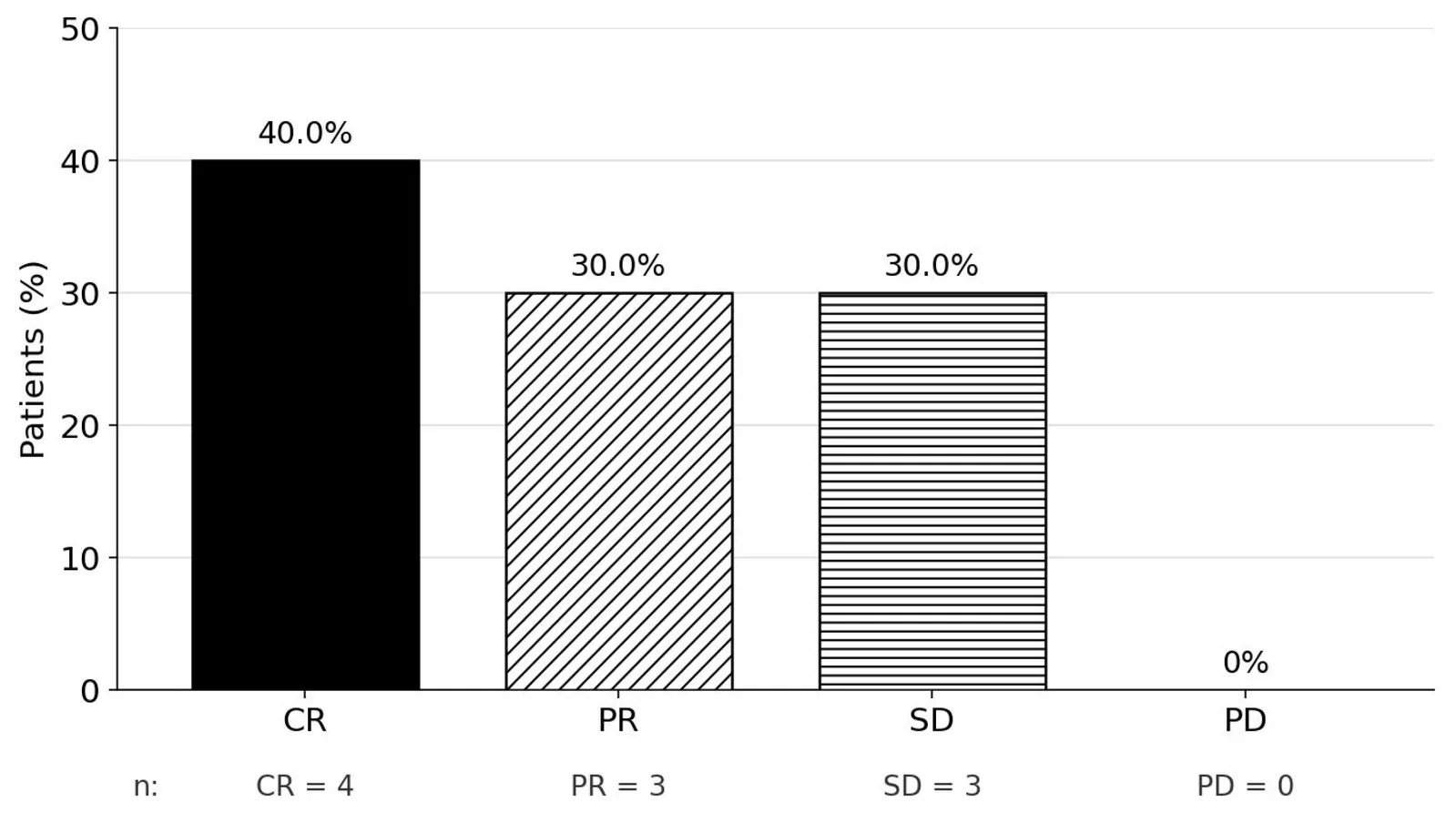
Investigator-assessed best overall response., CR complete response-; PR partial response; SD stable disease; PD progressive disease.

Out of 12 treated patients, 10 were evaluable for response (Figure 3). At a median follow-up of 11.6 months (range, 3.3 to 40.2 months), treatment discontinuation was attributed to disease progression (*n* = 4), adverse events (*n* = 3), therapy cost (*n* = 1), and patient preference (*n* = 2). Efficacy analyses demonstrated a confirmed complete response (CR) in 40.0% (*n* = 4) of patients and a partial response (PR) in 30.0% (*n* = 3), yielding an overall response rate (ORR) of 70.0% (95% CI, 34.8% to 93.3%) (Figure 4). The complete response (CR) rate observed with pharmacologic-dose SLM in combination with axitinib and pembrolizumab (36–40%) appears numerically higher than CR rates reported with contemporary first-line ICI-based combinations, including pembrolizumab plus axitinib (~10%), pembrolizumab plus lenvatinib (16.1%), nivolumab plus cabozantinib (13.9%), and nivolumab plus ipilimumab (12.0%). These cross-trial comparisons are descriptive only; given the small number of response-evaluable patients (*n* = 10) and the predominance of favorable- and intermediate-risk IMDC disease, and without adjustment for baseline differences, they should be interpreted cautiously as hypothesis-generating and warrant prospective randomized validation.

**Figure 5:**
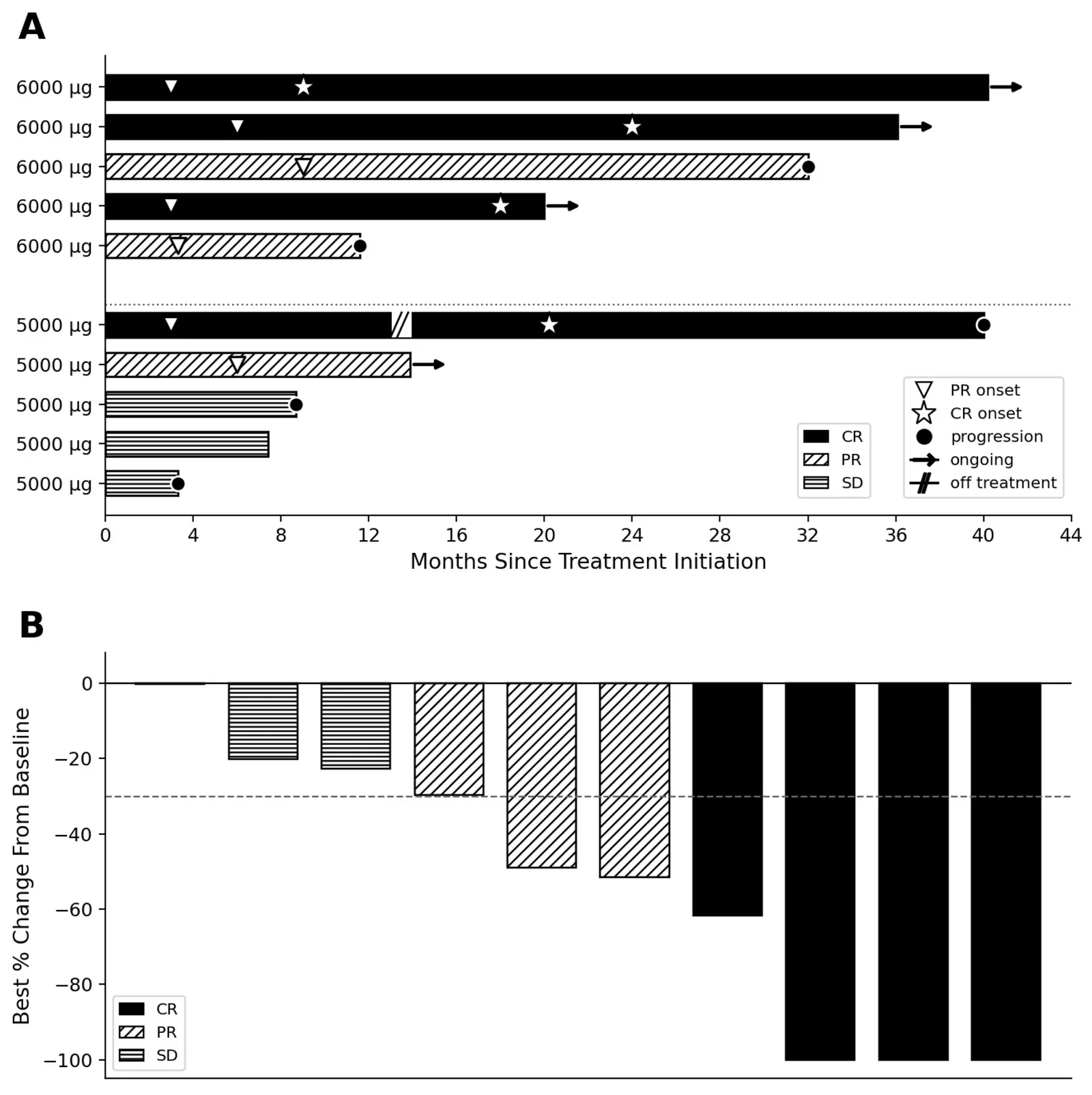
Clinical responses to SLM followed by axitinib plus pembrolizumab in metastatic ccRCC (RECIST v1.1; *n* = 10 response-evaluable). (**A**) Swimmer plot of time on treatment, grouped by SLM dose and shaded by best overall response; open triangles indicate partial-response onset, stars complete-response onset, arrows ongoing response, and × progression (patient 302, 5000 µg, came off treatment [break in bar] and later recurred; nodal CR shown). (**B**) Waterfall plot of best percentage change in target lesions from baseline, shaded by best overall response (dashed line, −30%). CR, complete response; PR, partial response; SD, stable disease; Patient 302 is classified as a nodal CR.

Stratification of the complete responses revealed an even split across International Metastatic Renal Cell Carcinoma Database Consortium (IMDC) risk categories, comprising two favorable-risk and two intermediate-risk patients. Long-term disease evaluation confirmed the durability of these responses, showing three ongoing CRs and two ongoing PRs. Longitudinal changes in the sum of target lesion diameters relative to secondary efficacy endpoints are detailed in Figure 5A,B. Kinetics of response varied notably by depth and time: while partial responses demonstrated an earlier onset—typically emerging between 4 and 8 months from the initiation of therapy—complete responses manifested as delayed therapeutic events, occurring significantly later in the treatment course. Toxicity subgroup analysis among the seven patients who experienced grade 3 hypertension revealed a diverse spectrum of clinical activity, comprising three CRs, two cases of stable disease (SD), and two instances of disease progression (PD). All therapeutic responses were independently verified and documented by investigators.

The principal finding of this study is that pharmacologic-dose seleno-L-methionine (SLM), administered in combination with axitinib and pembrolizumab, was associated with time-dependent pharmacodynamic suppression of the TGF-β1 axis, modulation of circulating immune/inflammatory mediators, and substantial clinical activity in previously untreated metastatic ccRCC. Three observations are particularly notable: (1) progressive suppression of circulating TGF-β1; (2) modulation of CXCL13, TNF-α, IFN-γ, IL-6, IL-8, and soluble PD-L1,;and (3) a 40% complete response (CR) rate. The CR rate compares favorably with the 11.6% CR rate reported with axitinib plus pembrolizumab in the 5-year KEYNOTE-426 analysis [4]. Although cross-trial comparisons cannot establish superiority, the convergence of pharmacodynamic, immunologic, and clinical findings provides a rationale for prospective evaluation of SLM as a resistance-modifying component of ICI/TKI therapy.

A particularly important pharmacodynamic observation was the progressive, time-dependent inhibition of circulating TGF-β1. Suppression was greater after approximately three months of continuous treatment with SLM in combination with axitinib and pembrolizumab than after the initial 14-day SLM-only exposure. Because axitinib and pembrolizumab were added on day 15, the greater suppression at the later time point reflects the combined regimen rather than prolonged SLM exposure alone, and the relative contribution of each agent cannot be determined in this single-arm study. This observation is consistent with the recently published phase I study of SLM plus axitinib, which demonstrated time-dependent increases in plasma selenium, TGF-β1 inhibition, and tumor vascular stabilization [7]. In that study, SLM was administered at 4000 μg twice daily for 14 days followed by 4000 μg once daily with axitinib 5 mg twice daily. Among 27 patients treated at the 4000-μg dose level, the objective response rate was 55.6%, median duration of response was 18.4 months, and median progression-free survival was 14.8 months [7].

The present study extends these observations to treatment-naïve metastatic ccRCC receiving SLM together with axitinib and pembrolizumab. TGF-β1 represents a biologically attractive upstream target because it integrates multiple processes implicated in ccRCC resistance, including immune suppression, stromal remodeling, angiogenesis, and metabolic adaptation. Preclinical studies further support this interpretation. Rataan et al. demonstrated direct interaction of methylseleninic acid (MSA) with TGF-β1 and showed that selenium treatment reduced TGF-β1, PD-L1, and VEGF in ccRCC models in a dose- and time-dependent manner [6,9,10,11]. These findings provide mechanistic context for the TGF-β1 suppression observed clinically.

The accompanying changes in CXCL13, TNF-α, IFN-γ, IL-6, IL-8, and soluble PD-L1 provide additional evidence of systemic biological activity. These mediators participate in interconnected processes involving immune-cell recruitment, activation, inflammation, and checkpoint regulation [13,14,15,16,17]. CXCL13 contributes to immune-cell recruitment and organization, whereas TNF-α and IFN-γ are important mediators of T-cell and NK-cell effector function [18,19,20]. In RCC, T-cell activation has been associated with increased IFN-γ and TNF signaling [21,22], whereas T-cell exhaustion has been linked to reduced TNF-α and IFN-γ, with the restoration of T-cell function accompanied by increased effector cytokines, granzyme B, and perforin [23]. Thus, the observed changes in TNF-α and IFN-γ are consistent with altered systemic immune effector activity. However, circulating cytokine concentrations should not be interpreted as direct surrogates for immune activity within the tumor microenvironment.

Importantly, the timing of biomarker assessment provides a potential explanation for the apparent separation between cytokine and TGF-β1 responses. Cytokines were measured at baseline and Day 14, whereas the greatest TGF-β1 suppression was observed after approximately three months of treatment with SLM in combination with axitinib and pembrolizumab. The Day 14 cytokine profile may therefore represent an early phase of immune remodeling rather than the maximal pharmacodynamic effect of sustained combination treatment. This temporal relationship is potentially important for future trial design and supports longitudinal assessment of TGF-β1 and immune mediators beyond the initial two weeks of treatment. Correlating these changes with tumor response, CR, and duration of response may help determine whether pharmacodynamic suppression of TGF-β1 can serve as a biomarker of therapeutic benefit.

Hypertension remains an important pharmacodynamic consequence of VEGFR inhibition by axitinib [24,25,26,27,28,29]. VEGF blockade can impair nitric oxide-mediated vasodilation and promote endothelial dysfunction and microvascular rarefaction, thereby increasing vascular resistance and blood pressure [8,28]. The Grade 3 hypertension observed with SLM plus axitinib and pembrolizumab is therefore consistent with potent VEGFR inhibition. Whether SLM contributes independently to vascular remodeling or modifies the vascular effects of axitinib remains uncertain. The absence of Grade 4 hypertension in this cohort is reassuring, but the vascular effects of the combination warrant prospective investigation.

The depth and timing of clinical response are additional clinically relevant observations. A 40% CR rate in this pilot cohort is substantially higher than the 11.6% CR rate reported with pembrolizumab plus axitinib in the 5-year KEYNOTE-426 analysis [4]. Notably, patients were able to remain on treatment without dose-limiting toxicity, while complete responses appeared to be delayed therapeutic events, emerging later during treatment rather than predominantly during the initial treatment period. This temporal pattern is consistent with the possibility that sustained treatment with the combination may progressively modify the tumor microenvironment over time. However, this comparison is exploratory and cannot establish superiority: with only 10 response-evaluable patients; a study population composed predominantly of favorable- and intermediate-risk IMDC disease; and no ability to adjust for differences in baseline characteristics, study design, follow-up, or response assessment, the cross-study comparison should be regarded as hypothesis-generating only. Nevertheless, the magnitude and timing of the responses raise the hypothesis that SLM may influence not only the probability of response but also the depth, timing, and durability of tumor regression.

A potential explanation for this activity is the complementary pharmacology of the three agents. Axitinib inhibits VEGFR-dependent angiogenic signaling, pembrolizumab restores antitumor T-cell activity through PD-1 blockade, and SLM acts upstream by the simultaneous inhibition of both a TGF-β1- and HIF-driven immunometabolic resistance network. The complementary vascular stabilization and immune modeling, together with selective enhancement of drug delivery, immune cell trafficking and tumor cell infiltration [6,7,10,29], provide a mechanistic basis for selective enhancement of the efficacy of SLM in combination with axitinib and pembrolizumab.

The innovation of this therapeutic concept therefore lies not simply in adding selenium to an established ICI/TKI regimen, but in using pharmacologic-dose SLM as a potential resistance-modifying agent targeting an upstream immunometabolic state. Rather than directly inhibiting a single downstream effector, SLM may simultaneously influence interconnected TGF-β1/HIF signaling, angiogenic pathways, immune checkpoints, and tumor metabolism. Such pathway-level modulation could complement axitinib-mediated vascular inhibition and pembrolizumab-mediated T-cell reactivation. The progressive nature of TGF-β1 suppression further suggests that the pharmacologic effect of SLM may evolve with continued exposure, providing a rationale for maintaining treatment rather than viewing SLM solely as a short-term sensitizing agent.

### 2.6. Limitations of the Study

Several limitations should be acknowledged. This was a small, single-arm-center pilot study and was not powered to establish definitive associations between biomarker modulation and clinical outcomes. Cytokine analyses were exploratory, and circulating biomarkers may not accurately reflect changes within the tumor microenvironment. Moreover, cytokines were evaluated primarily at an early time point, whereas maximal TGF-β1 suppression occurred later. The absence of paired tumor biopsies also prevented direct assessment of intratumoral TGF-β1, HIF signaling, PD-L1, VEGF, and metabolic pathways. Accordingly, the proposed mechanistic relationships remain hypothesis-generating rather than causal. Larger randomized studies incorporating longitudinal blood sampling, paired tumor pharmacodynamic analyses, and correlation of biomarker changes with response and survival will be required to validate this model.

In conclusion, this study provides preliminary clinical, pharmacodynamic, and immunologic evidence that pharmacologic-dose SLM modulates the TGF-β1 axis and systemic immune milieu when combined with axitinib and pembrolizumab in previously untreated metastatic ccRCC. Progressive TGF-β1 suppression and changes in CXCL13, TNF-α, IFN-γ, IL-6, IL-8, and soluble PD-L1 support biological activity beyond a single molecular target. Together with emerging clinical and preclinical evidence [6,7], these findings support randomized studies to determine whether SLM can reprogram immunometabolic resistance and enhance the depth and durability of ICI/TKI responses in ccRCC.

## 3. Materials and Methods

### 3.1. Study Design and Patient Treatment

This trial was registered at ClinicalTrials.gov (NCT05363631). The translational analyses reported here were performed on samples obtained from patients enrolled in an investigator-initiated, single-institution Phase I/II study of seleno-L-methionine (SLM) in sequential combination with axitinib and pembrolizumab in locally advanced and metastatic clear cell renal cell carcinoma (ccRCC) (University of Iowa, Iowa City, IA, USA; IND 126767). The study was approved by the institutional review board, and all patients provided written informed consent. Dose escalation of SLM was conducted using a standard 3 + 3 design at two dose levels—5000 µg (dose level 0) and 6000 µg (dose level 1). SLM was administered orally twice daily for 14 days as a priming (run-in) phase and continued once daily thereafter until disease progression or unacceptable toxicity. Beginning on day 15, axitinib (5 mg orally twice daily) and pembrolizumab (200 mg intravenously every 3 weeks) were added at standard doses and schedules [4,30]. SLM dosing was selected to achieve target whole-blood selenium concentrations of 35–45 µM, previously shown to be molecularly effective in xenograft models [13]. Twelve patients were treated, six at 5000 µg and six at 6000 µg. Correlative endpoints comprised whole-blood selenium concentration, plasma cytokine levels, and circulating (plasma) TGF-β1.

Eligible patients were adults (≥18 years) with histologically and radiologically confirmed locally advanced (unresectable) or metastatic clear cell RCC who were treatment-naïve in the metastatic setting; prior immunotherapy in the adjuvant setting was permitted. At least one non-irradiated RECIST v1.1 target lesion, an Eastern Cooperative Oncology Group performance status of 0–1, and adequate organ function were required, including hematologic (absolute neutrophil count ≥ 1.0 × 10^9^/L, platelets ≥ 100 × 10^9^/L, hemoglobin ≥ 7.0 g/dL), hepatic (AST/ALT < 3× the upper limit of normal [ULN], or <5× ULN with liver metastases; total bilirubin ≤ 1.5× ULN), and renal function (serum creatinine within institutional limits or creatinine clearance > 15 mL/min/1.73 m^2^ by Cockcroft–Gault), with adequately controlled blood pressure (≤150/90 mmHg). Prior cytoreductive nephrectomy was not required for eligibility. Key exclusions included untreated central nervous system metastases, an interfering prior or concurrent malignancy, a recent major cardiovascular event or major surgery, and the need for strong CYP3A4 inhibitors or inducers. All enrolled patients were treatment-naïve in the metastatic setting (no prior systemic lines in the metastatic setting; Table 1).

Patients were enrolled at a single institution (University of Iowa) between September 2022 and November 2025. The phase I primary endpoint was safety, assessed as dose-limiting toxicities and treatment-related adverse events graded by CTCAE v5.0; the phase II primary endpoint was the objective response rate (ORR; complete plus partial response) by RECIST v1.1. Secondary endpoints were progression-free survival and overall survival. Prespecified correlative (translational) endpoints comprised whole-blood selenium concentration, plasma cytokine modulation, and circulating (plasma) TGF-β1.

Treatment was administered in 21-day cycles and patients were evaluated in clinic at screening, on days 1 and 15 of the SLM run-in, and on day 1 of each subsequent 21-day cycle (i.e., approximately every 3 weeks, coincident with pembrolizumab administration), with interim safety telephone contact on cycle 1 day 15; assessments at each visit included physical examination, performance status, adverse-event review, and laboratory studies. Anti-tumor activity was assessed radiographically by CT of the chest, abdomen, and pelvis (or MRI), with bone scans at investigator discretion, using RECIST v1.1. Baseline imaging was obtained within 28 days before the first dose of SLM, and follow-up imaging was repeated every 12 weeks (±5 days) beginning at week 12 (cycle 5, day 1); complete and partial responses were confirmed by two assessments obtained at least 4 weeks apart.

### 3.2. Plasma TGF-β1 Quantitation

To assess the in vivo pharmacodynamic effect of SLM on TGF-β1, circulating (plasma) TGF-β1 was measured in patients. Peripheral blood was collected at baseline (day 1), on day 14 (after the SLM priming phase), and after approximately 3 months of treatment with SLM in combination with axitinib and pembrolizumab; plasma was separated and stored at −80 °C until analysis. Plasma TGF-β1 was quantified as previously described [6]. Tumor-tissue TGF-β1 assessed by Western blot refers to the preclinical 786-O ccRCC xenograft model (Figure 1A); paired tumor biopsies were not obtained from patients in this study.

### 3.3. Whole-Blood Selenium Quantitation

Whole-blood selenium concentrations were quantitated by inductively coupled plasma mass spectrometry (ICP-MS; Thermo Scientific Element XR high-resolution instrument, Thermo Fisher Scientific, Bremen, Germany) according to CDC method DLS 3016.8 (Selenium in Whole Blood; SOP ANK DCD 1939). Samples were collected at baseline and on day 14 [31,32].

### 3.4. Plasma Cytokine Profiling

Plasma samples were collected at baseline and after 14 days of SLM treatment and stored at −80 °C until analysis. Cytokines and chemokines—including IFN-γ, IL-6, IL-8, TNF, and CXCL13—were measured using a MILLIPLEX Human Cytokine/Chemokine/Growth Factor Panel A/B custom plex according to the manufacturer’s instructions (Millipore Sigma, Burlington, MA, USA) [18,33].

### 3.5. Statistical Analysis

This pilot study was designed as a translational, hypothesis-generating investigation and all analyses were descriptive. No formal sample-size or power calculation was performed and no formal hypothesis testing was prespecified. Categorical outcomes, including the objective response rate and the complete-response rate, are summarized as proportions with exact (Clopper–Pearson) binomial 95% confidence intervals. Continuous variables are reported as medians with ranges. Efficacy was analyzed in the response-evaluable population (patients with at least one post-baseline RECIST v1.1 assessment) and safety in all treated patients. Changes in correlative measures (plasma selenium, cytokines, and TGF-β1) from baseline to on-treatment time points are presented descriptively; where shown, nominal *p*-values from paired nonparametric tests (Wilcoxon signed-rank) are exploratory and were not adjusted for multiple comparisons. Analyses were performed in Microsoft Excel (Microsoft Corp., Redmond, WA, USA; Microsoft 365).

## 4. Conclusions

### 4.1. Take-Home Message

**Figure 6:**
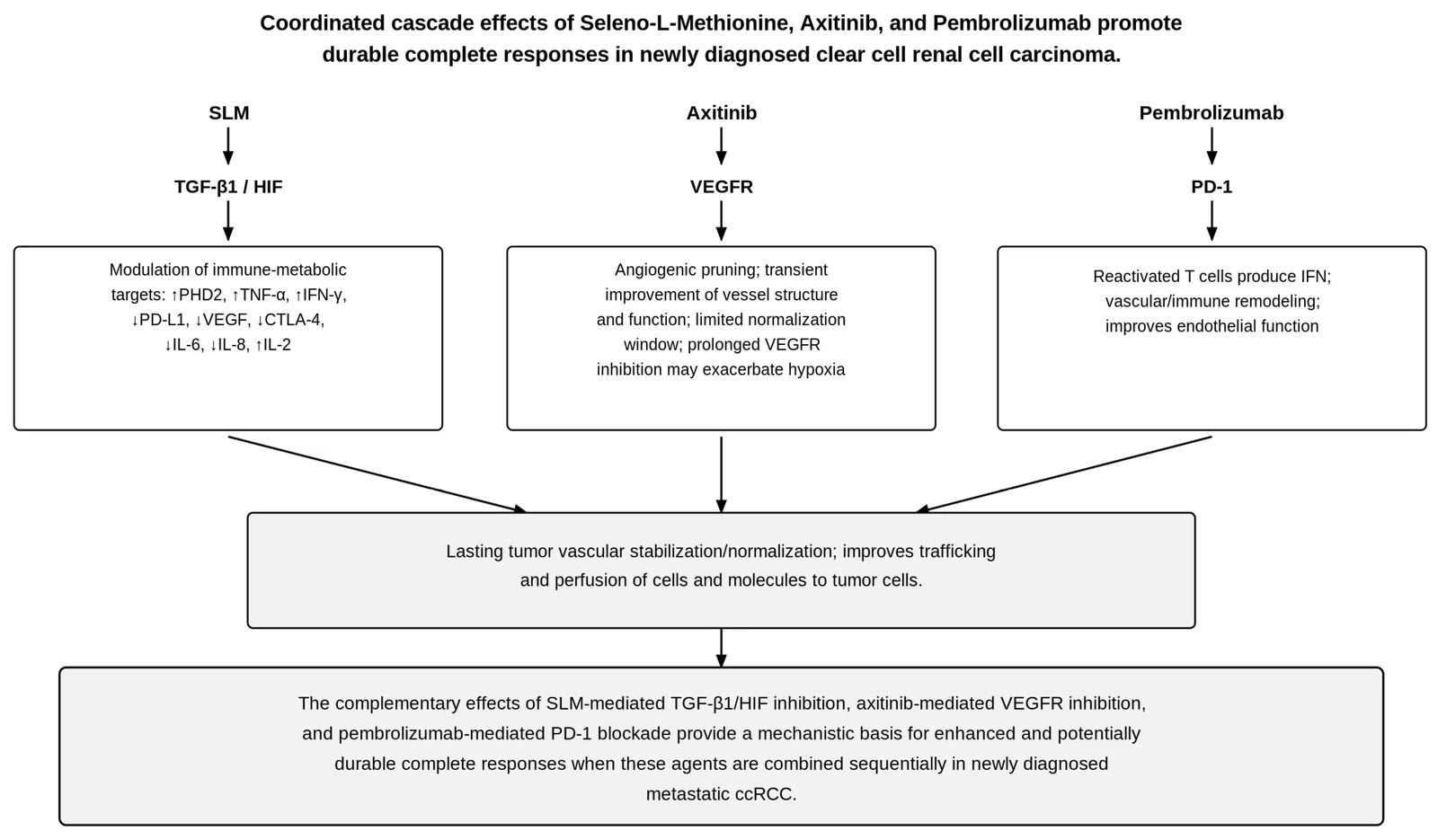
Coordinated cascade effects of seleno-L-methionine (SLM), axitinib, and pembrolizumab in newly diagnosed metastatic clear cell renal cell carcinoma. Each agent acts on a distinct upstream target—SLM on the TGF-β1/HIF axis, axitinib on VEGFR, and pembrolizumab on PD-1—producing complementary downstream effects that converge on lasting tumor vascular stabilization and normalization and on improved intratumoral delivery and immune-cell trafficking, providing a mechanistic basis for enhanced and potentially durable complete responses. Vertical arrows indicate the direction of the signaling cascade (each agent to its molecular target to the downstream effects, converging on the integrated outcome); upward (↑) and downward (↓) arrows denote increased and decreased expression or activity of the indicated mediators, respectively.

The preliminary SLM/axitinib/pembrolizumab experience demonstrated an overall response rate comparable to that reported with axitinib/pembrolizumab in KEYNOTE-426, but with a numerically higher proportion of complete responses. Given the small, nonrandomized sample and differences in patient populations, treatment characteristics, and follow-up, this observation is hypothesis-generating and requires prospective validation. A potentially important mechanistic distinction is the complementary effects of axitinib, pembrolizumab, and SLM on the tumor vasculature and microenvironment. As illustrated in Figure 6, axitinib directly inhibits VEGFR1–3 signaling, suppressing endothelial proliferation and vascular permeability and promoting regression and remodeling of abnormal tumor vessels. This can produce a transient window of vascular normalization; however, excessive or prolonged VEGFR inhibition may reduce vascular density and increase tumor hypoxia. Pembrolizumab, through PD-1 blockade, reactivates antitumor T cells and may indirectly improve vascular–immune interactions. In contrast, SLM targets upstream drivers of vascular dysfunction by inhibiting TGF-β1 and promoting the PHD2-dependent degradation of HIFs. This suppresses persistent proangiogenic and hypoxia-adaptive signaling, including VEGF expression, that contributes to abnormal and unstable tumor vasculature. Thus, whereas axitinib primarily produces relatively rapid VEGFR-dependent vascular remodeling and pruning, SLM may promote a more sustained state of vascular stabilization and normalization. The combination may therefore provide complementary rather than redundant vascular effects: axitinib suppresses pathological VEGF signaling and prunes abnormal vessels, pembrolizumab restores antitumor immune activity, and SLM suppresses the TGF-β1/HIF-driven microenvironment that sustains vascular instability and hypoxia. This coordinated remodeling may preserve a more functional tumor vasculature, potentially improving the intratumoral delivery of axitinib and immune-cell infiltration while reducing hypoxia-associated immune suppression. This mechanism provides a testable rationale for the observed clinical activity and supports the prospective evaluation of SLM as a tumor-microenvironment-modifying component of VEGF/PD-1-directed therapy.

### 4.2. Future Perspectives

Future clinical studies should incorporate integrated longitudinal translational endpoints, including serial assessment of plasma selenium, TGF-β1, HIF-regulated biomarkers, and inflammatory and immune-modulatory cytokines. Such an approach will be important for defining the pharmacodynamic effects of SLM, establishing target engagement, and identifying biomarkers predictive of response. In particular, the greater inhibition of the TGF-β1/HIF axis observed after prolonged treatment with SLM plus axitinib and pembrolizumab, compared with the early (SLM-only) treatment period, highlights the importance of longitudinal biomarker assessment and of trial designs capable of distinguishing the contribution of SLM from that of the combination. Serial assessment of CXCL13, TNFα, IL-6, IL-8, and soluble PD-L1, together with TGF-β1, may further clarify the temporal relationship between suppression of the TGF-β1/HIF axis, immune and inflammatory remodeling, and clinical response.

These findings warrant prospective clinical validation in an adequately powered study of the SLM, axitinib, and pembrolizumab triple combination to determine whether pharmacologic suppression of the TGF-β1/HIF axis translates into higher complete response rates, longer progression-free survival, and more durable clinical benefit compared with established ICI/VEGF TKI therapy.

The ability of SLM to suppress the TGF-β1/HIF signaling axis also suggests that its therapeutic potential may extend beyond the axitinib/pembrolizumab combination. Because this pathway contributes to tumor immune evasion, metabolic adaptation, abnormal angiogenesis, and therapeutic resistance, SLM could potentially serve as a broader immunometabolic conditioning strategy. Future studies should evaluate SLM in combination with emerging HIF-2α-directed therapies and explore its potential to enhance adoptive cellular therapies, including CD70- and Fn14-directed CAR-T cells [10,29]. This approach is supported by our preclinical findings demonstrating enhanced CAR-T-cell persistence and cytotoxicity following the selenium-mediated inhibition of TGF-β1/HIF signaling. Such studies could determine whether pharmacologic disruption of the TGF-β1/HIF axis can improve not only checkpoint-inhibitor and VEGF-targeted therapy but also cellular immunotherapy.

Ultimately, the clinical development of SLM should be guided by a biomarker-driven strategy linking drug exposure, sustained TGF-β1/HIF inhibition, cytokine remodeling, and clinical outcome. Establishing these relationships prospectively will be critical for determining whether the biological effects observed in this pilot study represent a reproducible mechanism of therapeutic enhancement and whether SLM can be developed as a broadly applicable immunometabolic adjunct to contemporary therapies for ccRCC.

## Figures and Tables

**Table 1:** Patient demographics and baseline characteristics of the efficacy population.

Variable	*n* = 12
**Age, median (min–max)**	71 (48–76)
**Sex, *n* (%)**	
Female	2 (16.7%)
Male	10 (83.3%)
**ECOG performance status**	
0	10 (83.3%)
1	2 (16.7%)
**Race or ethnic group, *n* (%)**	
White	12 (100.0%)
**IMDC risk group, *n* (%)**	
Favorable	4 (33.3%)
Intermediate	7 (58.3%)
Poor	1 (8.3%)
**Sarcomatoid features, *n* (%)**	0 (0.0%)
**Prior systemic therapies, *n* (%)**	
0	12 (100.0%)
1	0 (0.0%)
2+	0 (0.0%)

**Table 2:** AEs possibly, probably, or definitely related to study treatment present in >20% of the study population.

Toxicity	Total	Grade 1–2	Grade 3	Toxicity	Total	Grade 1–2	Grade 3
ALP increased	3 (25.0%)	3 (25.0%)	0 (0.0%)	Hyperglycemia	6 (50.0%)	6 (50.0%)	0 (0.0%)
ALT increased	8 (66.7%)	6 (50.0%)	2 (16.7%)	Hypertension	11 (91.7%)	4 (33.3%)	7 (58.3%)
AST increased	4 (33.3%)	3 (25.0%)	1 (8.3%)	Hyperthyroidism	4 (33.3%)	3 (25.0%)	1 (8.3%)
Abdominal pain	6 (50.0%)	4 (33.3%)	2 (16.7%)	Hypoalbuminemia	3 (25.0%)	3 (25.0%)	0 (0.0%)
Acute kidney injury	3 (25.0%)	1 (8.3%)	2 (16.7%)	Hyponatremia	6 (50.0%)	5 (41.7%)	1 (8.3%)
Anemia	3 (25.0%)	3 (25.0%)	0 (0.0%)	Hypotension	5 (41.7%)	4 (33.3%)	1 (8.3%)
Anorexia	8 (66.7%)	8 (66.7%)	0 (0.0%)	Hypothyroidism	4 (33.3%)	4 (33.3%)	0 (0.0%)
Arthralgia	5 (41.7%)	5 (41.7%)	0 (0.0%)	Insomnia	3 (25.0%)	3 (25.0%)	0 (0.0%)
Chills	3 (25.0%)	3 (25.0%)	0 (0.0%)	LDH increased	5 (41.7%)	5 (41.7%)	0 (0.0%)
Constipation	4 (33.3%)	4 (33.3%)	0 (0.0%)	Lymphocyte count decreased	4 (33.3%)	4 (33.3%)	0 (0.0%)
Cough	3 (25.0%)	3 (25.0%)	0 (0.0%)	Myalgia	4 (33.3%)	4 (33.3%)	0 (0.0%)
Creatinine increased	7 (58.3%)	6 (50.0%)	1 (8.3%)	Nausea	6 (50.0%)	6 (50.0%)	0 (0.0%)
Diarrhea	8 (66.7%)	6 (50.0%)	2 (16.7%)	PPE syndrome	6 (50.0%)	6 (50.0%)	0 (0.0%)
Dysgeusia	5 (41.7%)	5 (41.7%)	0 (0.0%)	Proteinuria	6 (50.0%)	6 (50.0%)	0 (0.0%)
Dyspnea	4 (33.3%)	4 (33.3%)	0 (0.0%)	Pruritus	4 (33.3%)	4 (33.3%)	0 (0.0%)
Edema, limbs	4 (33.3%)	4 (33.3%)	0 (0.0%)	Rash, acneiform	3 (25.0%)	3 (25.0%)	0 (0.0%)
Fatigue	10 (83.3%)	9 (75.0%)	1 (8.3%)	Rash, maculopapular	4 (33.3%)	4 (33.3%)	0 (0.0%)
Fever	5 (41.7%)	5 (41.7%)	0 (0.0%)	Thromboembolic event	5 (41.7%)	4 (33.3%)	1 (8.3%)
Headache	5 (41.7%)	5 (41.7%)	0 (0.0%)	Urinary tract infection	3 (25.0%)	2 (16.7%)	1 (8.3%)
Hemoptysis	3 (25.0%)	3 (25.0%)	0 (0.0%)	Vomiting	5 (41.7%)	5 (41.7%)	0 (0.0%)
Hoarseness	5 (41.7%)	5 (41.7%)	0 (0.0%)	Weight loss	7 (58.3%)	7 (58.3%)	0 (0.0%)

Values are *n* (%), counted once per patient at worst grade (CTCAE v5.0). Events occurring in ≥25% of patients shown. PPE = palmar–plantar erythrodysesthesia. No Grade 4–5 events observed.

## Data Availability

The data presented in this study are available on request from the corresponding author.

## Supplementary Materials

The following supporting information can be downloaded at: https://www.mdpi.com/article/10.3390/ijms27219825/s1.

## References

[1] Monjaras-Avila C.U., Lorenzo-Leal A.C., Luque-Badillo A.C., D’Costa N., Chavez-Muñoz C., Bach H. (2023). The tumor immune microenvironment in clear cell renal cell carcinoma. Int. J. Mol. Sci..

[2] Nguyen T.V., Hsu T. (2026). Immunometabolic stress and immune suppression in clear-cell renal-cell carcinoma: Perspectives in therapeutic strategy. Int. J. Mol. Sci..

[3] Tsai Y.C., Tsai V.S., Lin C.H., Kuo C.Y. (2026). Natural products in clear cell renal cell carcinoma: Rewiring the VHL–HIF axis, metabolic plasticity, and tumor–immune interactions. Int. J. Mol. Sci..

[4] Rini B.I., Plimack E.R., Stus V., Gafanov R., Waddell T., Nosov D., Pouliot F., Alekseev B., Soulières D., Melichar B. (2025). Pembrolizumab plus axitinib versus sunitinib for advanced clear cell renal cell carcinoma: 5-year survival and biomarker analyses of the phase 3 KEYNOTE-426 trial. Nat. Med..

[5] Vlachostergios P.J. (2020). Resistance to pembrolizumab and axitinib in renal cell carcinoma: Clinical and genomic evaluation. J. Kidney Cancer VHL.

[6] Rataan A.O., Xu Y., Geary S.M., Zakharia Y., Kamel E.S., Rustum Y.M., Salem A.K. (2025). Targeting transforming growth factor-β1 by methylseleninic acid/seleno-L-methionine in clear cell renal cell carcinoma: Mechanisms and therapeutic potential. Cancer Treat. Res. Commun..

[7] Zakharia Y., Reis R.J., Kroll M.R., Rataan A.O., Manchkanti S., Rahim B., Garje R., Swami U., Mott S.L., Zamba K. (2025). Phase I clinical trial of high doses of seleno-L-methionine in combination with axitinib in patients with previously treated metastatic clear cell renal cell carcinoma. Clin. Cancer Res..

[8] Yin M.B., Li Z.R., Tóth K., Cao S., Durrani F.A., Hapke G., Bhattacharya A., Azrak R.G., Frank C., Rustum Y.M. (2006). Potentiation of irinotecan sensitivity by Se-methylselenocysteine in an in vivo tumor model is associated with downregulation of cyclooxygenase-2, inducible nitric oxide synthase, and hypoxia-inducible factor 1α expression, resulting in reduced angiogenesis. Oncogene.

[9] Chintala S., Toth K., Cao S., Durrani F.A., Vaughan M.M., Jensen R.L., Rustum Y.M. (2010). Se-methylselenocysteine sensitizes hypoxic tumor cells to irinotecan by targeting hypoxia-inducible factor 1α. Cancer Chemother. Pharmacol..

[10] Rustum Y.M., Rataan A.O., Zakharia Y., Davila M., Amissah O.B., Kim G. (2026). Pharmacologic doses of ascorbic acid and seleno-L-methionine are a new and novel class of transforming growth factor-β1 inhibitor: Mechanisms and therapeutic potential. Med. Res. Arch..

[11] Zakharia Y., Bhattacharya A., Rustum Y.M. (2018). Selenium targets resistance biomarkers enhancing efficacy while reducing toxicity of anti-cancer drugs: Preclinical and clinical development (review). Oncotarget.

[12] Cao S., Durrani F.A., Rustum Y.M. (2004). Selective modulation of the therapeutic efficacy of anticancer drugs by selenium-containing compounds against human tumor xenografts. Clin. Cancer Res..

[13] Hirano T. (2021). IL-6 in inflammation, autoimmunity and cancer. Int. Immunol..

[14] Perez-Penco M., Byrdal M., de la Torre L.L., Ballester M., Khan S., Siersbæk M., Lecoq I., Madsen C.O., Kjeldsen J.W., Svane I.M. (2025). The antitumor activity of TGFβ-specific T cells is dependent on IL-6 signaling. Cell. Mol. Immunol..

[15] Gocher A.M., Workman C.J., Vignali D.A.A. (2022). Interferon-γ: Teammate or opponent in the tumour microenvironment?. Nat. Rev. Immunol..

[16] Aghaei M., Bahreiny S.S., Mahdizade A.H., Abbasi A., Magharati A., Yousefi-Avarvand A. (2026). Cytokine-mediated tumor sensitization: Mechanistic frameworks and therapeutic opportunities in cancer immunotherapy. Clin. Exp. Med..

[17] Kment J., Newsted D., Young S., Vermeulen M.C., Laight B.J., Greer P.A., Lan Y., Craig A.W. (2024). Blockade of TGF-β and PD-L1 by bintrafusp alfa promotes survival in preclinical ovarian cancer models by promoting T effector and NK cell responses. Br. J. Cancer.

[18] Zhao L., Jin S., Wang S., Zhang Z., Wang X., Chen Z., Wang X., Huang S., Zhang D., Wu H. (2024). Tertiary lymphoid structures in diseases: Immune mechanisms and therapeutic advances. Signal Transduct. Target. Ther..

[19] Teillaud J.L., Houel A., Panouillot M., Riffard C., Dieu-Nosjean M.C. (2024). Tertiary lymphoid structures in anticancer immunity. Nat. Rev. Cancer.

[20] Mempel T.R., Lill J.K., Altenburger L.M. (2024). How chemokines organize the tumour microenvironment. Nat. Rev. Cancer.

[21] Sirc N., Smolander J., Kumari A.N., Liu J., Lee M.H., Lahdensuo K., Järvinen R., Tornberg S.V., Lin L., Laitinen H.K. (2026). Functionally characterizing the renal cell carcinoma tumor-immune microenvironment via patient-derived ex vivo models. Cancer Res. Commun..

[22] Borcinova M., Bartolini R., Foley L.K., Novak V., Taborska P., Stakheev D., Rataj M., Smrz D., Fialova M., Hacek J. (2024). Distinct leukocyte populations and cytokine secretion profiles define tumoral and peritumoral areas in renal cell carcinoma. Transl. Oncol..

[23] Dai Z., Zhou H., Feng Z., Zhang M., Ai Z., Huang G., Cen J., Liang Y., Wei J., Chen W. (2026). Sialylation-immune-related lncRNA LINC01605 promotes tumor-infiltrating CD8+ T cell exhaustion and malignancy of clear cell renal cell carcinoma. Front. Immunol..

[24] Cohen J.B., Brown N.J., Brown S.A., Dent S., van Dorst D.C.H., Herrmann S.M., Lang N.N., Oudit G.Y., Touyz R.M. (2023). Cancer therapy-related hypertension: A scientific statement from the American Heart Association. Hypertension.

[25] Babu A.D., Singh S., Thota A., Duhan S., Sainatham C., Gill H., Raghavakurup L., Tantry U., Bliden K., Gurbel P. (2024). Systemic hypertension with VEGFR inhibitor (axitinib) therapy in cancer patients: A meta-analysis and systematic review of randomized controlled trials. Int. J. Cardiol. Heart Vasc..

[26] Pandey A.K., Singhi E.K., Arroyo J.P., Ikizler T.A., Gould E.R., Brown J., Beckman J.A., Harrison D.G., Moslehi J. (2018). Mechanisms of VEGF (vascular endothelial growth factor) inhibitor-associated hypertension and vascular disease. Hypertension.

[27] Motzer R.J., Escudier B., Tomczak P., Hutson T.E., Michaelson M.D., Negrier S., Oudard S., E Gore M., Tarazi J., Hariharan S. (2013). Axitinib versus sorafenib as second-line treatment for advanced renal cell carcinoma: Overall survival analysis and updated results from a randomised phase 3 trial. Lancet Oncol..

[28] Bhattacharya A., Seshadri M., Oven S., Toth K., Vaughan M., Rustum Y.M. (2008). Tumor vascular maturation and improved drug delivery induced by methylselenocysteine leads to therapeutic synergy with anticancer drugs. Clin. Cancer Res..

[29] Kim G.B., Amissah O.B., Ager C., Zakharia Y., Rustum Y.M. Methylseleninic acid enhances anti-FN14 CAR-T cell effector function and redirects cytotoxicity against TGF-β-rich kidney, prostate, and brain tumors. Proceedings of the AACR Annual Meeting.

[30] Rini B.I., Plimack E.R., Stus V., Gafanov R., Hawkins R., Nosov D., Pouliot F., Alekseev B., Soulières D., Melichar B. (2019). Pembrolizumab plus axitinib versus sunitinib for advanced renal-cell carcinoma. N. Engl. J. Med..

[31] Gardiner P.H., Littlejohn D., Halls D.J., Fell G.S. (1995). Direct determination of selenium in human blood serum and plasma by electrothermal atomic absorption spectrometry. J. Trace Elem. Med. Biol..

[32] Jones D.R., Jarrett J.M., Tevis D.S., Franklin M., Mullinix N.J., Wallon K.L., Quarles C.D., Caldwell K.L., Jones R.L. (2017). Analysis of whole human blood for Pb, Cd, Hg, Se, and Mn by ICP-DRC-MS for biomonitoring and acute exposures. Talanta.

[33] McKinski K., Tang H., Wang K., Birchler M., Wright M. (2025). Comparison of highly sensitive, multiplex immunoassay platforms for streamlined clinical cytokine quantification. Bioanalysis.

