# Supplementary material for: TGF-β1 Inhibition, Cytokine Modulation, and Clinical Response to Seleno-L-Methionine Plus Axitinib and Pembrolizumab in Treatment-Naïve Clear Cell Renal Cell Carcinoma
Source: Int J Mol Sci. 2026 Nov 2;27(21):9825. doi: 10.3390/ijms27219825 (PMC13763053; doi:10.3390/ijms27219825)
Supplement: Supplementary file 1 [file ijms-27-09825-s001.zip › Supplementary Materials.pdf]

Supplementary Materials

The following supporting information is provided: Figure S1 (plasma selenium dose–response).

Plasma Selenium Concentrations are SLM Dose-Dependent

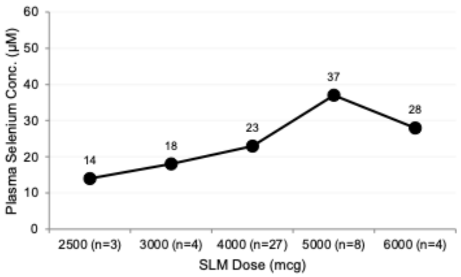

Figure S1. Plasma selenium concentrations in ccRCC patients treated with 2500, 3000, 4000, 5000, and 6000 µg SLM twice daily for 14 days, then concurrently with axitinib and pembrolizumab. Selenium was measured on day 14, 2 h after each SLM dose. Plasma selenium increased in a dose-dependent manner and was significant up to 5000 µg SLM, with no evidence of selenium-attributable host toxicity. These data are pooled from and derived in part from the prior phase I dose-escalation cohort [7].
